# Antimicrobial therapy approaches in the mastitis control driven by one health insights

**DOI:** 10.29374/2527-2179.bjvm002624

**Published:** 2024-07-17

**Authors:** Miliane Moreira Soares de Souza, Felipe Carlos Dubenczuk, Dayanne Araújo Melo, Thérèsse Camille Nascimento Holmström, Marcela Barlette Mendes, Elina Beatriz Reinoso, Shana Mattos Oliveira Coelho, Irene Silva Coelho

**Affiliations:** 1 Veterinarian, DSc., Departamento de Microbiologia e Imunologia Veterinária (DMIV), Instituto de Veterinária (IV), Universidade Federal Rural do Rio de Janeiro (UFRRJ). Seropédica, RJ, Brazil.; 2 Veterinarian, DSc. Programa de Pós-Graduação em Ciência e Tecnologia Agrária (PPGCTIA), DMIV, IV, UFRRJ. Seropédica, RJ, Brazil.; 3 Veterinarian, DSc., Programa de Pós-Graduação em Ciências Veterinárias (PPGCV), DMIV, IV, UFRRJ. Seropédica, RJ. Brazil.; 4 Animal Science, DSc., PPGCV, DMIV, IV, UFRRJ. Seropédica, RJ. Brazil.; 5 Veterinarian, Msc. PPGCV, DMIV, IV, UFRRJ. Seropédica, RJ. Brazil.; 6 Veterinarian, Dsc., Universidad Nacional De Río Cuarto, Córdoba, Argentina.; 7 Biologist, DSc., DMIV, IV, UFRRJ. Seropédica, RJ. Brazil.; 8 Agronomist, DSc., DMIV, IV, UFRRJ. Seropédica, RJ. Brazil.

**Keywords:** dairy production, WHO, superbugs, mastitis, produção leiteira, OMS, superbactérias, mastite

## Abstract

The use of antimicrobials in the dairy production environment for mastitis control must take etiology, clinical signs, economic impacts, and regulatory frameworks into consideration. The objective of the present review is to highlight important aspects of the dynamics of antimicrobial use in dairy production and the potential impacts on the main pathogens circulating in this environment, considering the parameters set by the World Health Organization (WHO) regarding the priority of monitoring as well as control strategies for these agents, such as the methicillin-resistant *Staphylococcus* and the beta-lactamase-producing *Escherichia coli*. Understanding the animal-environment-pathogen triad is crucial for establishing control measures and preventing the spread of bacterial resistance. Implementing mastitis prevention and control measures in dairy farms, considering process flow and personnel qualification, enables a reduction in antimicrobial usage and contributes to prevent the spread of resistant bacterial agents in the dairy production environment, minimizing the relapses and the chronicity of the infectious process.

## Resumo

A utilização de antimicrobianos no controle de mastite em ambiente de produção leiteira deve considerer alguns aspectos como a etiologia, os sinais clínicos, os impactos económicos e a legislação. O objetivo da presente revisão é destacar aspectos importantes na dinâmica do uso de antimicrobianos na produção leiteira e os potenciais impactos sobre os principais patógenos circulantes neste ambiente, considerando os parâmetros estabelecidos pela Organização Mundial da Saúde (OMS) quanto à prioridade de monitoramento, bem como estratégias de controle para esses agentes, como o *Staphylococcus* resistente à meticilina e a *Escherichia coli* produtora de beta-lactamase. Compreender a tríade animal-ambiente-patógeno é crucial para estabelecer medidas de controle e prevenir a propagação da resistência bacteriana. A implementação de medidas de prevenção e controle de mastites nas propriedades leiteiras, considerando o fluxo do processo e a qualificação do pessoal, permite a redução do uso de antimicrobianos e contribui para prevenir a propagação de agentes bacterianos resistentes no ambiente de produção leiteira, minimizando as recidivas e a cronicidade do processo infeccioso.

## Introduction

The use of antimicrobials in the dairy production environment for mastitis control must take etiology, clinical symptoms, economic impacts, and regulatory frameworks into consideration. Understanding the animal-environment-pathogen triad is crucial for implementing control measures and preventing the spread of resistant bacteria (Economou & Gousia, 2015; Van Boeckel et al., 2015). The decrease in milk production and alterations in its composition are consequences of changes associated with the severity and extent of the inflammatory process, which are closely tied to the characteristics of the causative agent involved (More et al., 2022). Implementing mastitis prevention and control measures in dairy farms, considering process flow and personnel qualification, enables the reduction in antimicrobial usage and contributes to prevent the spread of resistant bacterial agents in the dairy production environment, minimizing the relapses and the chronicity of the infectious process (More et al., 2022; Ajose et al., 2022).

Mastitis, the mammary gland inflammation, is a multifactorial process that may have physiological, traumatic, mechanical, chemical or infectious origins (Ajose et al., 2022; Cheng & Han, 2020; Cobirka et al., 2020; More et al, 2022; Ruegg, 2021). Bacterial mastitis is the most common form in dairy cows, and the intensity of the inflammatory response is influenced by the virulence of the pathogen as well as by the cow's ability to mount a rapid and effective immune response (Cheng & Han, 2020). A mild inflammatory response leads to neutrophil influx in the gland without noticeable alterations, while a more severe response causes visible localized or generalized signs of inflammation (Cheng & Han, 2020; Cobirka et al., 2020).

Mastitis is classified according to three criteria: clinical presentation, duration of the infectious process, and mode of infection transmission (Ajose et al., 2022; Ruegg, 2021). Clinical mastitis is characterized by observable changes in the milk, signs of udder inflammation (edema, pain, heat, redness), and systemic symptoms like fever, dyspnea, anorexia, and prostration (Ruegg, 2021). Severity can be classified into three levels, based on the presence of these symptoms (Fredebeul-Krein et al., 2022). Grade 1 is characterized just by the changes in milk characteristics alone, Grade 2 involves milk alterations and signs of udder inflammation, and Grade 3 includes systemic symptoms such as fever, dyspnea, anorexia and prostration. Clinical mastitis can also be classified as hyperacute, acute or chronic, according to the onset time and duration of the signs (Fredebeul-Krein et al., 2022). Hyperacute and acute mastitis are characterized by rapid onset and intense but short-lived alterations, often caused by bacteria circulating in the production environment (Jamali et al., 2018). Chronic mastitis is associated with fibrosis of the mammary tissue and permanent changes in milk quality. Antimicrobial therapy is recommended for clinical mastitis cases caused by pathogens with a low rate of spontaneous cure, while high therapeutic cure index pathogens may not require antimicrobial treatment due to the potential for spontaneous resolution (Fredebeul-Krein et al., 2022; Jamali et al., 2018; Ruegg, 2021).

Subclinical mastitis, defined by the Somatic Cell Count in milk, does not present visible signs of inflammation or fibrosis but can lead to a reduction in milk quality and quantity. Treatment with a therapeutic protocol during lactation is usually not recommended for subclinical mastitis (Gonçalves et al., 2018).

Mastitis is traditionally classified as either environmental or contagious, based on the pathogen transmission (Cheng & Han, 2020; Cobirka et al., 2020, Morales-Ubaldo et al., 2023). Environmental mastitis occurs when cows come into direct contact with environmental pathogens, primarily bacteria, present in the surrounding environment (Cheng & Han, 2020; Cobirka et al., 2020; Morales-Ubaldo et al., 2023). These pathogens enter the udder through the teat canal, which remains open for a period ranging from one or two minutes up to two hours after milking (Cheng & Han, 2020; Cobirka et al., 2020). In contagious mastitis transmission occurs during milking, through infected mammary quarters, by the milkers' hands, and in milking unit liners as fomites. Environmental mastitis is commonly caused by Enterobacterales (e.g., *Escherichia coli*, *Klebsiella* spp., *Serratia* spp.) (Bronzato et al., 2017; Rodrigues et al., 2017) and other opportunistic bacteria (e.g., Coagulase-Negative *Staphylococcus* (CNS), *Streptococcus uberis*, *Streptococcus dysgalactiae*, *Lactococcus* spp., *Trueperella pyogenes*, *Pseudomonas* spp.) (Abureema et al., 2014; Melo et al., 2018; Morales-Ubaldo et al, 2023; Soares et al., 2017), as well as by fungi, yeasts, and algae like *Prototheca* spp. (Osumi et al., 2008). Contagious mastitis is associated with *Staphylococcus aureus*, *Streptococcus agalactiae*, *Corynebacterium bovis* and *Mycoplasma* spp (Morales-Ubaldo et al., 2023).

Recent advancements in diagnostic tools have led to a more accurate understanding of mastitis epidemiology, challenging the strict environmental or contagious classification. Some authors argue that bacteria like *S. aureus* and *Str. agalactiae*, traditionally classified as contagious pathogens, can also be considered environmental pathogens based on their transmission patterns (Abureema et al., 2014). Indeed, the transmission of pathogens like *S. aureus* and *Str. agalactiae* can occur through various routes, including contaminated bedding, feces, urine and other fomites, contradicting this strictly contagious classification. Additionally, there are differing opinions regarding the classification of *Prototheca* as a causative agent of environmental mastitis (Osumi et al., 2008).

Understanding the different models of pathogen dissemination is crucial for implementing the appropriate mastitis control protocol. Measures such as milking hygiene, disinfection and dry cow therapy have proven to be more effective to control the transmission of contagious mastitis. *S. aureus* remains a significant challenge for herds without effective control strategies, but implementing pro*Gram*s focused on udder health, sanitary management (including hygienic milking), therapeutic protocols for treating clinical mastitis, and drying cows with mastitis can significantly reduce its impact (Alencar et al., 2014).

## Diagnosis of mastitis

The diagnosis of mastitis is based on clinical observations or procedures to measure the inflammatory response, since the invasion of the glandular tissue by microorganisms triggers the arachidonic acid cascade and the complement system (Kour et al., 2023). The inflammatory response in the mammary gland is primarily composed of Polymorphonuclear Neutrophils (PMN) cells, and their migration to milk can be detected within three hours of the inflammation onset (Ezzat Alnakip et al., 2014; Oviedo-Boyso et al., 2007). The intensity of the response is often indicated by an increase in the Somatic Cell Count (SCC) and depends on the virulence of the pathogen as well as on the immune status of the host. A mild inflammatory response leads to neutrophil influx in the gland without noticeable changes, while a more severe response causes visible localized symptoms or generalized signs of inflammation (Safak & Risvanli, 2022).

It is important to emphasize that inflammation signs do not necessarily indicate an active bacterial infection, and the diagnosis of clinical mastitis should involve identifying the causal agent through phenotypic or molecular methods (Cheng & Han, 2020). These methods are also useful for understanding the bacterial agents that are circulating in dairy farms and to determine the therapeutic protocol to be adopted, along with prevention strategies able to reduce the incidence and prevalence of mastitis in the herd (Kour et al., 2023).

The Somatic Cell Count (SCC) is a diagnostic test used to detect subclinical mastitis and can serve as an indicator of udder health (Safak & Risvanli, 2022; Zhang et al., 2022). Cows with a threshold SCC of less than 200,000 cells/mL are considered healthy or recovered from mastitis, while those with SCC higher than 200,000 cells/mL are considered to have an intramammary infection (Zhang et al., 2022). In a healthy, uninfected mammary gland, milk typically contains macrophages, PMN, and lymphocytes in quantities that do not exceed 50,000 cells/mL. However, in clinical mastitis cases, depending on the characteristics of the causative agent, SCC threshold can significantly increase and reach rates greater than 5,000,000 cells/mL (Safak & Risvanli, 2022). Since milk from cows with subclinical mastitis often appears to be visibly normal, it is mixed with milk from healthy animals for sale, as treating subclinical mastitis during lactation is generally not recommended, except in cases caused by *Streptococcus agalactiae*, where "blitz" therapy is recommended, requiring the treatment of all affected animals (Ruegg, 2021; Abureema et al., 2014).

## Economic and social impact of mastitis on the dairy herd

Among the economic losses resulting from mastitis are the decrease in milk quality and quantity, the premature culling of affected animals, and an increase in expenses related to treatment and veterinary care (Cheng & Han, 2020; Dejyong et al., 2022). The milk production reduction in the affected mammary gland is due to secretory tissue damage and capillary permeability changes with the consequent impairment of the synthesis capacity (Novac & Andrei, 2020). This can result in decreased levels of milk constituents like fat, casein and lactose, as well as an increase in blood-derived elements such as albumin, immunoglobulins, defense cells, chloride, sodium and free fatty acids. These alterations renders the milk unsuitable for consumption along with the production of derivatives, leading to its rejection (He et al., 2020; Oviedo-Boyso et al., 2007).

In 2022, Brazilian production was higher than 34 billion liters/year, being the third largest producer in the world, behind United States of America (USA) and India (Brasil, 2023) Despite being the third largest producer of milk in the world, Brazil's herd productivity is significantly lower than that of the USA as a consequence of various factors, including low genetic potential, inadequate management, unskilled labor, technological limitations, inefficient economic policies and health problems such as mastitis. These factors contribute to a lower milk production per cow and a lower overall productivity in the Brazilian dairy industry (Rocha et al., 2020). 

Economic losses caused by mastitis are not exclusive to Brazil. Recent data from different countries such as Egypt, China and the USA demonstrate the significant costs associated with mastitis, including treatment expenses, milk disposal, decreased productivity and potential culling of the affected animals. In Egypt (2020), the cost per clinical case of mastitis considering treatment and milk disposal was estimated at US$ 83.88 (Azooz et al., 2020). In China, estimated losses due to clinical mastitis were $12,000-76,000 per farm/month, equivalent to approximately $29-35 per cow/year (He et al., 2020). In the USA, losses have been estimated at approximately 180-200 dollars per cow/year. Based on the estimated costs of preventing and managing mastitis in Brazil, the total loss for the dairy industry, considering a dairy herd of around 9.5 million cattle, is estimated to be around US$ 1.8 to 2.0 billion. The cost of preventing mastitis was estimated at US$ 23.98/cow/year, and losses due to subclinical mastitis averaged at US$ 317.38/cow/year. The total cost of mastitis prevention on average for the producer was estimated at US$ 1558.59/dairy herd/year. Costs to producers attributable to cases of subclinical mastitis were estimated to average US$20,611/dairy herd/year (USDA, 2023).

After a 50-year gap, the Brazilian regulatory frameworks for milk quality and its derivatives have been constantly updated in the last two decades. Normative Instructions 51 (09/18/2002) (Brasil, 2002) and 62 (12/29/2011) (Brasil, 2011) of the Ministry of Agriculture, Livestock and Food Supply (MAPA) established stricter indexes for Somatic Cell Count, total bacterial count, and detection of antimicrobial residues in milk in accordance with the global standards (Brasil, 2018a, 2018b).

The publication of the Normative Instructions (NI) 76 and 77 (11/26/2018) further expanded the quality criteria from the production to the pasteurized product. NI 76 establishes the identity and quality characteristics that refrigerated raw milk, pasteurized milk, and type A pasteurized milk must have, including analyses aimed at detecting preservatives and antimicrobial residues. NI 77 emphasizes the need for self-control pro*Gram*s focused on the health status of the herd, milk suppliers' qualification, selection and training pro*Gram*s for transporters, registration systems for transporters and producers, including georeferencing (Brasil, 2018a).

## Antimicrobial use in the treatment of mastitis

The Journal of Dairy Science published a centenary edition in 2017 that presented a timeline going back to 1917, a pre-antibiotic era where little could be done about intramammary infections (Ruegg, 2017). Measures for controlling the transmission of *Streptococcus agalactiae* were limited to periodic milk examination, segregation, and slaughter of the affected cows (Plastridge et al., 1936). The introduction of the oral administration of sulfanilamide therapy had a significant impact in these paradigms. However, establishing effective drug concentrations in milk or blood and eliminating udder *streptococci* remained troublesome (Ruegg, 2017). In 1956, Murphy emphasized the futility of relying solely on treating clinical mastitis and highlighted the importance of sanitary management to address the root causes. Despite the limited understanding and questionable effectiveness of the methods used to reduce new infections, the indiscriminate application of antimicrobial therapy for both lactating and dry cows became widely adopted for mastitis treatment (Murphy, 1956).

In the late 1960s, the need to evaluate individual parameters such as age, number of lactations, productivity and severity of infection in the decision-making process regarding antimicrobial use was first stressed in a publication by Philpot (1969). Nevertheless, even decades later, these recommendations still lack recognition for their importance in property sanitary management. Noteworthy researchers in the field have emphasized that only animals within a selected profile are able to respond to antimicrobial therapy (Caneschi et al., 2023).

Even a century later, mastitis remains the most frequent bacterial infection in dairy production, since its treatment and prevention account for most antimicrobials administered to adult cows (Bhakat et al., 2020; Ruegg, 2017). While the limitations of antimicrobial use in mastitis treatment are known, it still represents the best protocol for controlling *Streptococcus agalactiae* due to its location in the mammary gland's duct system (Abureema et al., 2014; Ruegg, 2021). However, *S. aureus*'s ability to penetrate the duct wall and establish numerous *foci* leads to significant therapeutic failures, representing an even more considerable challenge than the high profiles of antimicrobial resistance presented by this bacteria species (Coelho et al., 2011).

Recently, *Gram*-negative bacteria mastitis has emerged as a problem, instigating research on treatment protocols, particularly for acute and hyperacute cases (Bronzato et al., 2017; Rodrigues et al., 2017; Sharun et al., 2021). In the past, antimicrobial classes for *Gram*-negative cells were limited, resulting in the use of drugs like chloramphenicol, which is now banned for lactating cows (Lees et al., 2021). The indiscriminate use of antimicrobials is a concern for both the consumers and the health authorities in view of the advances in the understanding of One Health, sustainability and animal welfare concepts. Currently, the rational and balanced use of antimicrobials is a subject of global studies (Souza et al., 2020).

The primary reason for administering antimicrobials to dairy cows is to treat mastitis, therefore, the responsible use of these drugs is important (Cheng & Han, 2020; Li et al., 2023; Rodriguez et al., 2024; Rollin et al., 2015). The adoption of a therapeutic protocol for mastitis must consider various aspects, including hygienic-sanitary management measures, mastitis characterization based on etiology, clinical symptoms, economic impacts, regulatory frameworks, among others (Rodriguez et al., 2024).

The costs associated with the diagnosis, milk disposal, medication dilution and elimination in the subsequent milking, as well as with the low bacteriological cure rate (not exceeding 50%) in staphylococcal infections make treating subclinical mastitis during lactation uneconomical (Li et al., 2023; Rodriguez et al., 2024; Rollin et al, 2015). Meanwhile, "blitz" therapy, which involves mapping all cases of mastitis on a property and treating all cows with mastitis caused by *Streptococcus agalactiae* during lactation, has been shown to be economically viable when accompanied by prophylactic measures aimed at controlling mastitis levels on the property and preventing new infections (Abureema et al., 2014; Rollin et al., 2015; Ruegg, 2021).

Dry cow therapy, which involves treating subclinical mastitis at the end of the lactation period, uses specific pharmaceutical products formulated with antimicrobials that have slow elimination and absorption properties (Niemi et al., 2021; Rowe et al., 2023). The goal is to cure existing mastitis during the drying process (Niemi et al., 2021; Rowe et al., 2023; Scherpenzeel et al., 2016). However, in some cases, intramammary antimicrobials are questionably administered in healthy quarters to prevent new infections during the dry period (Müller et al., 2023). Dry cow therapy not only minimizes the issue of antimicrobial residues in milk but also yields better results when compared to treating lactating cows (Niemi et al., 2021; Rowe et al., 2023; Scherpenzeel et al., 2016). For farms with high management and control, selective therapy is recommended, targeting high-risk cows or those affected with mastitis during drying. Although there is no difference in cure rates or postpartum outcomes, selective therapy is shown to reduce antimicrobial use in the production system by up to 60% (Müller et al., 2023; Ruegg, 2018).

It is important to follow a therapeutic protocol for clinical mastitis based on the severity of the condition, which may include anti-inflammatories, antimicrobials, supportive therapy and bacteriological analysis of milk samples (Jong et al., 2023; Sharun et al., 2021; Timonen et al., 2021). The intensity of the clinical symptoms will guide the protocol to be implemented. Mild to moderate mastitis cases usually require administering anti-inflammatories and conducting a bacteriological analysis of the milk samples to determine the appropriate treatment. (Ruegg, 2018; Svennesen et al., 2023; Wilm et al., 2021). In severe mastitis cases, antimicrobials (intramammary and systemic), anti-inflammatory drugs (preferably non-steroidal), supportive therapy (oral hydration and intravenous hypertonic solution), and bacteriological analysis of milk samples are recommended to prevent septicemia or toxemia, since these processes are mostly caused by *Gram*-negative rods of the order Enterobacterales such as *E. coli* (Ruegg, 2018, 2021).

Before introducing antimicrobial therapy for non-severe clinical mastitis, it is essential to determine the infectious nature, causative agent, origin, dissemination source and susceptibility profile to ensure an effective treatment (Sharun et al., 2021). The prognosis must also be established, considering both the probability of therapeutic cure and the possibility of systemic conditions leading to animal death (Ruegg, 2018; Sharun et al., 2021). Determining the causative agent is important because structural differences in the bacterial cell wall result in distinct antimicrobial susceptibility profiles. This is particularly relevant for *Gram*-negative bacteria, which are intrinsically resistant to many antimicrobials used to treat mastitis (Bronzato et al., 2017; Rodrigues et al., 2017; Ruegg, 2018).

Despite these well-known parameters, empirical treatment without considering specific factors is commonly adopted without specialized advice, neglecting the clinical diagnosis, animal history, etiological agent, and even in cases of negative culture (Ajose et al., 2022; Ruegg, 2018, 2021). As a result, all cases of mastitis are treated with the same therapeutic protocol, regardless of etiology and potential consequences (Ajose et al, 2022; Ruegg, 2018, 2021). In practice, most clinical mastitis cases are not treated according to recommended guidelines, such as using the intramammary administration route, with the appropriate products and adhering to technical specifications. Instead, the indiscriminate use of antimicrobials via intramuscular, intravenous or subcutaneous routes is more common. This can result in relapses, chronicity, selection of resistant agents, and contribute to the spread of antimicrobial resistance in dairy units (Jong et al., 2023; Ruegg, 2018; Sharun et al., 2021; Svennesen et al., 2023; Timonen et al., 2021; Wilm et al., 2021).

In the past decade, there has been a shift towards prioritizing prevention over antimicrobial treatments in the guidelines for controlling bovine mastitis (Gerber et al., 2021; Kour et al., 2023; Ruegg, 2018). However, it is crucial to provide professional training to veterinarians, zootechnicians, and agricultural technicians to effectively communicate and implement proper hygienic-sanitary management along with preventive measures to the individuals working in the field. This will facilitate the establishment of efficient disease control methods that encompass not only individual animals but the entire herd (Alencar et al, 2014; Gerber et al, 2021).

The recent implementation of on-farm diagnosis is a helpful strategy for producers and technicians focused on milk quality (Griffioen et al., 2021; Vasquez et al., 2018). When combined with well-known strategies like the dark bottom mug test, it provides necessary screening for the correct implementation of therapeutic protocols aimed at controlling clinical mastitis in the herd of each property (McDougall et al., 2018). In addition to the microbiological aspects, it is necessary to analyze which cows may benefit from treatment according to various criteria such as being the first case of mastitis, having less than 3 lactations, having less than 3 teats with mastitis, and not having chronic mastitis (previous SCC threshold must be less than 200,000) (Cheng & Han, 2020; Safak & Risvanli, 2022; Wilm et al, 2021).

When the preliminary analysis indicates that treatment may not be beneficial, the cow should be isolated, and early drying off should be considered with inactivation of the affected mammary quarter and milk disposal until full recovery (Zigo et al., 2021). Upon considering a therapeutic protocol with antimicrobials for treating clinical mastitis, it is recommended to collect a milk sample for a microbiological culture after identifying the clinical case, preferably during the first milking (Ruegg, 2018, 2021). Following these principles, it is possible to contemplate the implementation of the following treatment protocols:

- For non-*aureus Staphylococcus* and *Streptococcus agalactiae*, it is recommended to use intramammary cephalosporins or semi-synthetic penicillins (ceftiofur, cefquinome, cephalexin, cefapirin, ampicillin, amoxacillin, cloxacillin) for 3 days (Lago et al., 2016; Sharun et al., 2021; Timonen et al., 2021). For environmental *Streptococcus*, the treatment duration should be from 5 to 6 days (Lago et al., 2016; Ruegg, 2018);
- For *Staphylococcus aureus*, the low cure rate associated with this agent must be considered. However, if a therapeutic protocol is adopted, it is recommended to use intramammary cephalosporins or cloxacillin for 8 days (Jong et al., 2023; Lago et al., 2016);
- For *Klebsiella* spp., which also has a low cure rate, it is recommended to follow the same treatment logic as with *S. aureus*. If a therapeutic protocol is adopted, it is recommended to use intramammary cephalosporins for 5 to 6 days (Ruegg, 2018, 2021; Sharun et al, 2021);
- For *Escherichia coli* and other *Gram*-negative bacteria *(Enterobacter* spp., *Pseudomonas* spp.), *Prototheca*, and yeasts, it is not recommended to adopt a protocol with antimicrobials for clinical mastitis in grades 1 and 2 (Sharun et al., 2021). Instead, supportive and anti-inflammatory treatments should be used to reduce the clinical symptoms (Blackmon et al., 2024). *E. coli* has a high rate of spontaneous cure, and the other agents are refractory to intramammary antimicrobials (Ruegg, 2018).

In cases of a negative culture result, it is not recommended to proceed with the treatment, since there is a high possibility of bacteriological cure, with only signs of inflammation remaining (Schmenger & Krömker, 2020). Nevertheless, it is important to note that the applied methodology may not identify certain agents, such as *Mycoplasma* spp (Parker et al., 2018). It is crucial to avoid making empirical alterations to the treatment protocol, such as extending the treatment duration or changing the antimicrobial agent used (Ruegg, 2018). There is no evidence to support that altering the therapeutic protocol improves cure rates for animals that were not cured in the previously adopted protocol (Ruegg, 2018).

## Pharmacological characteristics of antimicrobials for intramammary use

When treating mastitis in lactating animals using the Intramammary Route (IMM), it is desirable that the medicine persists for a short period of time in the gland, as it reduces the presence of residues in the milk after the treatment, favoring release for consumption. Since milk is an aqueous suspension, there is a preference for vehicles that aid the diffusion of the antimicrobial agents, such as polyvinylpyrrolidone (Alves et al., 2023). Conversely, the treatment of animals during the drying period requires long persistence of antimicrobials, which entails the use of vehicles such as the combination of mineral oil with 3% aluminum monostearate, a salt derived from stearic acid and aluminum that is used in gel formulations (Souza & Dubenczuk, 2022). This combination reduces the solubility of the antimicrobial agent, thereby increasing its effectiveness in the mammary gland. Additionally, pharmaceutical products for cows during the dry period contain higher concentrations of one or more antimicrobials (McCubbin et al., 2022).

Pharmacokinetics takes the physicochemical properties of drugs into consideration, as they affect the concentration that reaches the gland (Souza & Dubenczuk, 2022). For instance, antimicrobials injected through the teat canal diffuse rapidly and can reach the entire gland, while being absorbed from the udder into the bloodstream and vice versa, depending on the degree of protein binding and the ionization constant (pKa) of the drug (Souza & Dubenczuk, 2022). Pharmacodynamics is another important characteristic to consider, as it relates to the drug's interaction with the microorganisms within the target compartment. Antimicrobial activity within inflamed breast glandular tissue is different from normal glandular tissue, for example (Zhang et al., 2021). The pH of normal milk ranges from 6.4 to 6.8, making it slightly acidic. However, in the mammary gland with mastitis, due to increased vascular permeability, the pH becomes slightly alkaline, approaching the plasma pH of 7.2 to 7.4. When mastitis is caused by lactose-fermenting microorganisms, depending on their number within the gland, the pH may become more acidic or remain unchanged (Souza & Dubenczuk, 2022).

## Characteristics of antimicrobials for systemic use in the treatment of mastitis

In the treatment of mastitis, the intramuscular or subcutaneous route is typically preferred for administering antimicrobials, although in hyperacute cases the intravenous route may be necessary (Ruegg, 2022). Apart from the antimicrobial spectrum and potency, the apropriate treatment is influenced by a good drug distribution and diffusion in the breast tissue. The mammary gland has high blood perfusion, approximately 10 liters per minute. Adequate drug distribution through this parenteral route depends on factors such as lipid solubility, pKa, pH and protein binding (Ruegg, 2018).

From a pharmacological standpoint, an ideal antimicrobial for the systemic treatment of mastitis should have a broad spectrum of action and easily reach optimal concentrations in the gland without affecting other systems, such as the gastrointestinal tract (Ruegg, 2021). It should be highly liposoluble, with low plasma protein binding, and act as a weak base according to the slightly acidic pH of normal milk. It is important to note that the pH can be altered by the inflammatory process or microorganism. Additionally, an essential characteristic of an appropriate antimicrobial agent is maintaining activity in the presence of the inflammatory process (Zhang et al., 2021). Under normal conditions, penicillins, cephalosporins, aminoglycosides, and sulfa drugs do not have an efficient distribution in the mammary gland when systemically administered. On the other hand, macrolides (such as erythromycin and tylosin), tetracyclines, trimethoprim, and fluoroquinolones tend to exhibit a good distribution (Souza & Dubenczuk, 2022).

Several IMM antimicrobials can penetrate the mammary gland barriers, but most have a relatively limited spectrum of activity against *Gram*-positive bacteria (Ruegg, 2021). In the USA, dairy farmers have access to seven approved antimicrobial products for IMM use, including one lincosamide (pirlimycin) and six beta-lactams such as 1st (cefapirin) and 3rd (ceftiofur) generation cephalosporins, aminopenicillins (amoxicillin and hetacillin), penicillin G, and a penicillinase-resistant penicillin (cloxacillin), with no approved systemic product for treating clinical mastitis (Ruegg, 2022). In Brazil, the situation is significantly different. A survey identified approximately 20 active ingredients currently available in intramammary formulations, totaling around 50 different products. These include both broad and narrow spectrum antimicrobials, either as standalone or combination treatments. Similarly, a significant number of systemic products list mastitis as one of the targeted diseases. This diversity creates challenges for the identification and implementation of the most appropriate therapeutic protocol for each case, highlighting the importance of perfoming the microbiological evaluation as the foundation for making informed treatment choices (Souza & Dubenczuk, 2022).

## Antimicrobial residues in milk

The presence of contaminants in food is a major concern for food safety. Therefore, the presence of antimicrobial residues in dairy products is a significant issue, as it not only poses risks to consumer health but also disrupts derivative production, often rendering it unfeasible and resulting in substantial economic losses (Priyanka et al., 2017; Virto et al, 2022). Consumer health risks associated with antimicrobial residues in milk may include the development of antimicrobial resistance, hypersensitivity reactions (particularly with beta-lactam antibiotics like penicillins), carcinogenicity, mutagenicity, teratogenicity, bone marrow depression and alteration of the normal gut microbiome. Allergic reactions, including anaphylactic shock, can rapidly occur in sensitive individuals due to the presence of these residues (Jeena et al, 2020).

Effectively, the persistence of antimicrobial residues in milk is influenced by various factors, including dosage, administration route, excipients, solubility and drug combination (Priyanka et al., 2017; Virto et al, 2022). The intensity of the mammary gland inflammatory process also interferes with the elimination period of the antimicrobial residues after the treatment, be it either intramammary or systemic, often beyond the recommended withdrawal period (Sharun et al., 2021). It is crucial to ensure accurate information in antimicrobial package inserts, particularly regarding withdrawal periods during lactation, to prevent the presence of residues in milk (Priyanka et al, 2017).

The Codex Alimentarius, jointly developed by the Food and Agriculture Organization (FAO) and World Health Organization (WHO), establishes international standards for food safety. In Brazil, the NI 77 (11/26/2018) (Brasil, 2018b) establishes residue monitoring as part of the implementation of good agricultural practice protocol along with the milk suppliers' qualification (Brasil, 2018b). The official confirmatory test is High Performance Liquid Chromatography (HPLC) performed in accredited laboratories that are part of the Brazilian Network of Milk Quality Control Laboratories (RBQL) (Brasil, 2019a, 2019b).

## Dairy production and one health

The objective of the present review is to acknowledge the impact of dairy production on the spread of antimicrobial resistance due to the excessive use of antimicrobials, in an attempt to control bovine mastitis and ensure safe food production for human consumption. It is crucial to analyze how the dairy production environment contributes to the deterioration of health issues related to pathogen transmission through milk (Paramasivam et al., 2023), as well as the consequences of using subtherapeutic doses of antimicrobials for extended periods, creating favorable conditions for the circulation and persistence of resistance genes that can be transmitted to human-adapted pathogens (Souza et al., 2016).

*Streptococcus agalactiae* stands out as a major pathogen implicated in the etiology of contagious mastitis (Cheng et al., 2019). Despite its relevance in dairy production and the fact that its detection in the herd, even when associated with subclinical mastitis, warrants antimicrobial therapy (Rollin et al., 2015), this microorganism is not listed among the agents of greatest interest when considering a One Health approach based on the WHO list of priorities concerning antimicrobial resistance pattern (World Health Organization, 2017c). This highlights the need for a more comprehensive approach when addressing antimicrobial resistance, taking specific dairy industry-related factors into consideration (Salam et al., 2023).

Unlike *Staphylococcus aureus*, which exhibits persistence strategies such as biofilm formation and significant antimicrobial resistance, making its eradication from cattle herds can be extremely difficult (Marques et al., 2013, 2017). The circulation of S. *aureus* strains carrying resistance genes against the ß-lactam class is quite high, contributing to the widespread dissemination of the mostly known mechanisms for this resistance: production of ß-lactamases through *blaZ* gene expression and synthesis of altered penicillin-binding protein (PBP2a) encoded by the *mecA* gene, conferring methicillin resistance and representing an evolutionary shift in the dynamics of beta-lactam resistance in *Gram*-positive cocci (Melo et al., 2014; Mendonça et al., 2012; Silva et al., 2013; Soares et al., 2012, 2021; Souza et al., 2012).

Previous studies (Melo et al., 2014; Mendonça et al., 2012; Silva et al., 2013; Soares et al., 2012, 2021) have reported several phenotypic methicillin-resistant *Staphylococcus* spp isolates that are not correlated with the presence of the *mecA* gene. Melo et al. (2020) reported the discovery of a variant of the *mecA* gene in bovine samples, which contains mutations in the annealing region that prevent gene detection with the primers described so far. The increase in selection pressure can indeed promote the spread of resistance genes and the emergence of specific mutations, making it difficult to accurately analyze resistance in dairy production environments (Melo et al., 2020). It is important to note that many intramammary pharmaceuticals containing cloxacillin are ineffective against methicillin-resistant strains, which is often overlooked as a selection criterion (Fischer-Tenhagen et al., 2023). Considering the impact of methicillin-resistant *Staphylococcus aureus* on human health, it is crucial to assess the risks of using this class of antimicrobials in animal production without specific indication (Marques et al., 2017; Melo et al., 2018; Soares et al., 2012; Souza et al., 2016, 2020).

*Staphylococcus* spp. also poses a potential risk of transmission to humans through milk and dairy products, as some species can produce toxins, including enterotoxins produced by *S. aureus* and occasionally by CNS carrying *sec* genes (Abril et al., 2020). These thermostable enterotoxins are resistant to pasteurization and boiling, being able to cause food poisoning with a short incubation period and varying symptoms depending on individual susceptibility, with greater severity in newborns, the elderly and immunocompromised individuals. Staphylococci in raw milk can originate from cows with mastitis, milking hands, or poor hygienic management,as well as inadequate sanitary and storage conditions increase the risk to human health (Abril et al., 2020; Rall et al., 2014).

Most enterobacteria pathogens associated with human enteric diseases derive from animals and can be transmitted either directly to humans or indirectly, through animal-origin food, contaminated water or a common reservoir (Santiago et al., 2016). Currently, strains that produce β-lactamases have been found in animal production environments and animal-derived foods, suggesting a potential route for their spread across different ecosystems. *Gram*-Negative Fermenting rods (GNF) of the order Enterobacterales, particularly *Escherichia coli*, are particularly problematic for global antimicrobial resistance control due to their ability to produce a wide range of beta-lactamases (Silva & Lincopan, 2012). The detection of Extended-Spectrum β-Lactamases (ESBL) in bacteria from animals has raised concerns about the transmission of ESBL genes between humans and animals. *E. coli* strains carrying *AmpC*-β-lactamases have been found in both healthy and sick animals, including food-producing animals (Silva & Lincopan, 2012). In 2019, *AmpC*-hyperproducing *E. coli* was detected in dairy herds in Brazil, with no previous reports of these Antimicrobial Resistance (AMR) bacteria in dairy cattle. However, several mutation positions observed in *E. coli* from beef cattle, broiler and meat had already been described in human samples (Santiago et al., 2019). These findings suggest a potential transmission route for these bacteria in the food chain and their dissemination in the environment.

ESBL or plasmid-mediated *AmpC*-β-lactamase producers are often resistant to aminoglycosides and fluoroquinolones. The prevalence of resistance to these antibiotics among *E. coli* isolates from animals has been rising, and the impact of animal-derived Broad-Spectrum-β-Lactamase-producing *Gram*-negative bacteria on public health has gained significant global attention (Santiago et al., 2016, 2019; Silva & Lincopan, 2012). Following the introduction of litter in the shed, *Klebsiella* spp. has gained significant prominence as a pathogen affecting the mammary gland and is considered a priority bacterium in the context of worldwide resistance emergence Santiago et al. (2016, 2019), Song et al. (2023).

*Enterococcus* spp. is another important bacterial group in the etiology of mastitis according to the One Health perspective (Różańska et al., 2019). They are associated with long-term subclinical intramammary infections, frequent clinical episodes and low success rates of therapeutic protocols, as a consequence of their resistance to routinely used antimicrobials like semi-synthetic penicillins and cephalosporins (Różańska et al., 2019). *Enterococcus* spp. gained attention in public health after the emergence of vancomycin-resistant strains in poultry production associated to the use of avoparcin as a growth promoter in the past few decades (Ribeiro et al., 2023).

The contribution of animal production environments to the global antimicrobial resistance widespread requires integrated research efforts using different approaches (O’Neil, 2016; Souza et al., 2020). Evaluating factors that increase the selective pressure in different environments, including unjustified therapeutic approaches that create ideal conditions for the circulation and maintenance of resistance genes, is a complex and multifactorial task (Sharma et al., 2018; World Health Organization, 2015). The study of strategies aiming to reduce the selection pressure and disrupt the microorganism transmission cycles should consider analyzing the distribution of bacterial species, their genetic diversity, antimicrobial resistance profiles, and the detection of genetic elements responsible for the resistance mechanisms (Hoque et al., 2020). Understanding the role of the dairy production environment in bacterial resistance development and gene dissemination is critical for implementing mitigation measures that involve the following key changes (More et al., 2022):

- Restrictions on the prophylactic use of antimicrobials in animals to exceptional cases, for individual or limited animals, when the risk of infection is high and severe consequences are likely (World Health Organization, 2017a);
- Restriction on the metaphylactic use of antimicrobials in groups of animals (World Health Organization, 2017b);
- The possibility to reserve certain antimicrobials for human use only (O’Neil, 2016).

## Conclusions

The analysis of the contribution of animal production environments to the antimicrobial resistance widespread is a complex and multifactorial task that requires integrated research efforts.

Given that the main use of antimicrobials in the dairy production environment is to treat mastitis, its necessary to assure its correct diagnosis, since understanding the different models of pathogen dissemination is crucial for implementing effective mastitis control protocols. The advancements in diagnostic tools have significantly improved our understanding of mastitis epidemiology, and strategies such as the "blitz" therapy, which involves mapping all cases of mastitis on a property and treating all cows with mastitis caused by *Streptococcus agalactiae* during lactation, can be valuable in controlling mastitis levels and preventing new infections.

The implementation of on-farm diagnosis is also a helpful strategy that must be combined with the analysis of which cows would benefit from treatment, based on criteria such as being the first case of mastitis, having less than 3 lactations, having less than 3 teats with mastitis, and not having chronic mastitis.

Finally, the proposed alterations in therapy protocols, including restricting the prophylactic and metaphylactic use of antimicrobials, along with reserving last-resort antimicrobials for human use, are important steps toward combating antimicrobial resistance.
